# *Melissa officinalis* L. as a Nutritional Strategy for Cardioprotection

**DOI:** 10.3389/fphys.2021.661778

**Published:** 2021-04-22

**Authors:** Nevena Draginic, Vladimir Jakovljevic, Marijana Andjic, Jovana Jeremic, Ivan Srejovic, Marina Rankovic, Marina Tomovic, Tamara Nikolic Turnic, Andrey Svistunov, Sergey Bolevich, Isidora Milosavljevic

**Affiliations:** ^1^Department of Pharmacy, Faculty of Medical Sciences, University of Kragujevac, Kragujevac, Serbia; ^2^Department of Human Pathology, 1st Moscow State Medical University IM Sechenov, Moscow, Russia; ^3^Department of Physiology, Faculty of Medical Sciences, University of Kragujevac, Kragujevac, Serbia; ^4^Research Institute of Pharmacy, 1st Moscow State Medical, University IM Sechenov, Moscow, Russia

**Keywords:** *Melissa officinalis*, lemon balm, cardioprotection, myocardium, polyphenols

## Abstract

This review aimed to provide a summary on the traditional uses, phytochemistry, and pharmacological activities in the cardiovascular system and cardiotoxicity of *Melissa officinalis* (MO), with the special emphasis on the protective mechanisms in different cardiovascular pathologies. MO is a perennial aromatic herb commonly known as lemon balm, honey balm, or bee balm, which belongs to *Lamiaceae* family. Active components are mainly located in the leaves or essential oil and include volatile compounds, terpenoid (monoterpenes, sesquiterpenes, triterpenes), and polyphenolic compounds [rosmarinic acid (RA), caffeic acid, protocatechuic acid, quercitrin, rhamnocitrin, luteolin]. For centuries, MO has been traditionally used as a remedy for memory, cognition, anxiety, depression, and heart palpitations. Up until now, several beneficial cardiovascular effects of MO, in the form of extracts (aqueous, alcoholic, and hydroalcoholic), essential oil, and isolated compounds, have been confirmed in preclinical animal studies, such as antiarrhythmogenic, negative chronotropic and dromotropic, hypotensive, vasorelaxant, and infarct size–reducing effects. Nonetheless, MO effects on heart palpitations are the only ones confirmed in human subjects. The main mechanisms proposed for the cardiovascular effects of this plant are antioxidant free radical–scavenging properties of MO polyphenols, amelioration of oxidative stress, anti-inflammatory effects, activation of M2 and antagonism of β1 receptors in the heart, blockage of voltage-dependent Ca^2+^ channels, stimulation of endothelial nitric oxide synthesis, prevention of fibrotic changes, etc. Additionally, the main active ingredient of MO-RA, *per se*, has shown substantial cardiovascular effects. Because of the vastness of encouraging data from animal studies, this plant, as well as the main ingredient RA, should be considered and investigated further as a tool for cardioprotection and adjuvant therapy in patients suffering from cardiovascular diseases.

## Introduction

During the history, for centuries people have been widely using medicinal plants as remedies for various cardiovascular diseases (CVDs) such as congestive heart failure, hypertension, angina pectoris, atherosclerosis, cerebral insufficiency, venous insufficiency, and arrhythmia (Mashour et al., 1998). Nowadays, the number one cause of death worldwide belongs to CVDs, taking approximately 17.9 million lives each year (31%), and despite the wide spectrum of synthetic cardiovascular drugs, the prevalence of CVDs is still growing (World Health Organization, 2021). Because of this ongoing health, social, and economic problem, science has turned to investigation of alternative medicine and natural products that are marked as traditionally efficient and safe in the treatment of CVDs (Mashour et al., 1998; Li et al., 2015; World Health Organization, 2021). Popularity of natural products in the science field has revived interest in traditional remedies as new cardiovascular drugs.

**FIGURE 1:**
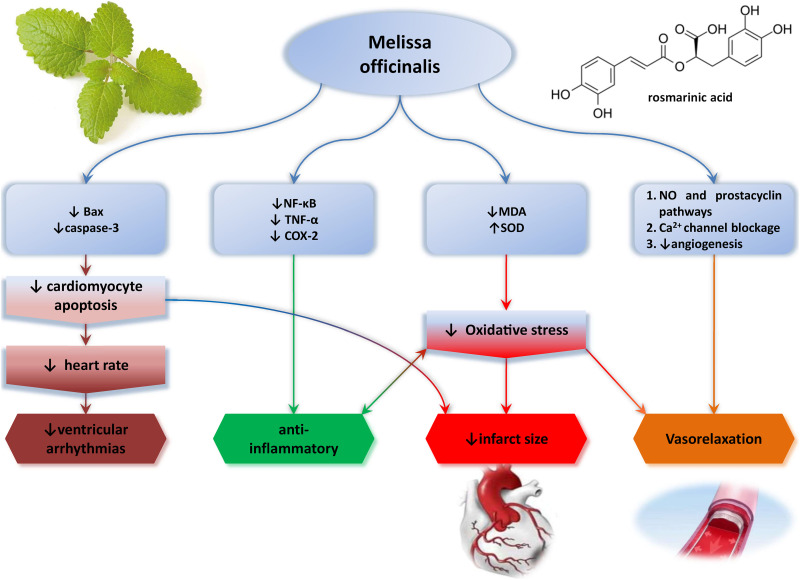
Cardioprotection induced by *Melissa officinalis*.

Despite the fact that the usage of *Melissa officinalis* (MO) in some cardiac pathologies was reported, up until now, there is a restricted knowledge regarding its cardioprotective potential and mechanisms involved. Thus, this review article aimed to summarize the current knowledge about MO effects on cardiovascular system and point out its possible usage as adjuvant therapy in CVD (Figure 1).

## Taxonomy

Kingdom: *Plantae—Plants*; subkingdom: *Tracheobionta—*vascular plants; superdivision: *Spermatophyta—*seed plants; division: *Magnoliophyta—*flowering plants; class: *Magnoliopsida—*dicotyledons; subclass: *Asteridae*; order: *Lamiales*; family: *Lamiaceae—*mint family; genus: *Melissa* L.*—*balm P; species: *Melissa officinalis* L.

## Botany

*Melissa officinalis* (Greek word “Melissa”—honeybee) is a perennial aromatic herb commonly known as lemon balm, honey balm, or bee balm. It belongs to *Lamiaceae* (mint) family of plants and is also a member of genus *Melissa* L. together with *Melissa axillaris*, *Melissa flava*, and *Melissa yunnanensis.* MO has square-shaped stem or quadrangular stem, which grows up to 0.5–1.5 m in height, characteristically for *Lamiaceae* members. The leaves are in decussate pairs, green ovate to cordate, and are used as herbal drug, because of the best active component content (Canadanović-Brunet et al., 2008; Abdel-Naime et al., 2016; Shakeri et al., 2016; Abdel-Naime et al., 2020). Also, the leaves possess lemon-like scent and taste due to the presence of volatile compounds (monoterpenes and sesquiterpenes) (Aharizad et al., 2012). During the summer, white/pink pale flowers are formed, whereas hairy root system includes several lateral roots (Canadanović-Brunet et al., 2008; Abdel-Naime et al., 2016, 2020; Shakeri et al., 2016). It is native to southern Europe and central and western Asia, especially the Mediterranean, but also in gardens and naturalized in many parts of the United States (Eastern, Midwestern, and Pacific Northwest states (Abdel-Naime et al., 2016, 2020).

## Active Compounds

MO is considered to be a great source of a wide range of active chemical compounds present in leaves or essential oil, including different terpene and polyphenolic compounds.

A vast number of studies investigated the MO essential oil content and claim that the major compounds of MO essential oil are volatile compounds such as monoterpenes and sesquiterpenes citrals (geranial and neral, which give citrus-like aroma), geraniol, citronellal, thymol, and β-caryophyllene in different proportions. The presence of additional components and their percentage vary, depending on the method of obtaining the essential oil, distillation conditions, region’s climate, plant species, and maturity stage of MO (Aharizad et al., 2012; Ehsani et al., 2017). Analysis of two essential oils obtained by hydrodistillation method from MO grown in Iran and Turkey discovered more than 20 different components and very similar content of the major components: citronellal (37.33% vs. 36.62–43.78%), thymol (11.96% vs. 0.40–11.94%), citral (10.10% vs. 10.10–17.43%), and β-caryophyllene (7.27% vs. 5.91–7.27%) (Cosge et al., 2009; Ehsani et al., 2017). On the other hand, MO essential oil obtained from Algeria is composed predominantly of oxygenated monoterpenes [neral (30.2%), geranial (44.2%) and citronellal (6.3%)], whereas the sesquiterpene fraction was lower: α-copaene (1.8%) and β-caryophyllene (1.3%) and oxygenated sesquiterpene caryophyllene oxide (1.3%) (Abdellatif et al., 2014). The essential oil of MO grown in Balkan, Serbia, was reported to be composed of mostly citrals (geranial + neral, 39.9%), citronellal (13.7%), limonene (2.2%), geraniol (3.4%), β-caryophyllene (4.6%), β-caryophyllene oxide (1.7%), and germacrene D (2.4%) (Mimica-Dukic et al., 2004). The variability in essential oil composition obtained from 15 different MO subspecies grown under Central European climate conditions was recently investigated, with the aim to compare the content between two harvests in June or August. It seems that leaves collected in June are richer in oil compared to the ones collected in August, whereas the oil content [dominantly citrals (geranial, neral) and citronellal] does not significantly differ between these two cuts (Chizzola et al., 2018). Plant age may also influence essential oil content, according to the recent investigation. Namely, high fluctuations in the content exist when comparing the first and the second year of growth, in the favor of richer chemical composition of the 2 year-old plant (Nurzyńska-Wierdak et al., 2014).

Phytochemical examinations of the MO leaves have pointed out the presence of the following main compounds: previously mentioned monoterpenes, sesquiterpenes (Mimica-Dukic et al., 2004; Cosge et al., 2009; Aharizad et al., 2012; Abdellatif et al., 2014; Nurzyńska-Wierdak et al., 2014; Ehsani et al., 2017; Chizzola et al., 2018), triterpenes, and phenolic compounds such as polyphenols, phenolic acids [rosmarinic acid (RA), caffeic acid, protocatechuic acid] (Ibragic et al., 2014), flavonoids (quercitrin, rhamnocitrin, luteolin) (Mencherini et al., 2007), and tannins. Rich phenolic content of lemon balm is mostly responsible for its therapeutic effects, due to well-known antioxidant potential of these compounds, so it is important to optimize the extraction conditions to achieve optimal phenolic content and therefore the treatment goals. Phenolic content also varies, depending on the plant origin. For example, hot water extracts of lemon balm leaves of Bosnian origin have higher content of rosmarinic, gallic, and chlorogenic acid compared to Turkish one, whereas the content of cinnamic acid was higher in Turkish origin lemon balm (Ibragic et al., 2014). The main triterpenoid compounds isolated from lemon balm are ursolic and oleanolic acid (Ibragic et al., 2014; Tantry et al., 2014), but additional two sulfated ursane-type triterpenes, one sulfated oleanane-type triterpene, and one oleanane triterpene were confirmed in the hydroalcoholic extract of the lemon balm leaves (Tantry et al., 2014). Triterpenes with sulfate groups in their structure, attached to the carbohydrate chain, are known to possess higher biological activity, relative to the one attached to the aglycone group (Park et al., 2014). Characteristically for Lamiaceae family, flavonoid compounds are mostly concentrated in the aerial parts of MO. Several flavonoid compounds have been isolated from lemon balm, divided into four groups by their chemical structure: flavones (luteolin, apigenin, and their derivatives), flavanones (hesperidin, hesperetin, naringin, naringenin), flavanols (catechin, epicatechin, rutin), and flavonols (isoquercitrin, rhamnocitrin) (Patora and Klimek, 2002; Mencherini et al., 2007; Dastmalchi et al., 2009; Pereira et al., 2014; Tantry et al., 2014; Shakeri et al., 2016). The main constituents are presented in Table 1.

**TABLE 1:** Major chemical constituents of *M. officinalis* essential oil and leaves.

*M. officinalis*	Essential oil	Leaf
Terpenes -Monoterpenes	Citronellal (Aharizad et al., 2012; Cosge et al., 2009; Ehsani et al., 2017) Thymol (Cosge et al., 2009; Aharizad et al., 2012; Ehsani et al., 2017) Citral (neral and geranial) (Cosge et al., 2009; Aharizad et al., 2012; Abdellatif et al., 2014; Ehsani et al., 2017)	
-Sesquiterpenes	β-Caryophyllene (Cosge et al., 2009; Aharizad et al., 2012; Abdellatif et al., 2014; Ehsani et al., 2017) Caryophyllene oxide (Mimica-Dukic et al., 2004; Abdellatif et al., 2014) Limonene (Mimica-Dukic et al., 2004) Germacrene D (Mimica-Dukic et al., 2004)	
-Triterpenes		Ursolic acid (Ibragic et al., 2014; Tantry et al., 2014) Oleanolic acid (Ibragic et al., 2014; Tantry et al., 2014) 3β,16β,23-Trihydroxy-13,28-epoxyurs-11- ene-3-O-β-D-glucopyranoside (Mencherini et al., 2007) 3,23-Disulfate ester of 3β,19α,23- trihydroxyurs-12-en-28-oic acid (Mencherini et al., 2007) 3,23-Disulfate ester of 2α,3β,19α,23- tetrahydroxyurs-12-en-28-oic acid (Mencherini et al., 2007) 3,23-Disulfate ester of 2α,3β,19α,23-tetrahydroxyurs-12-en-28-oic acid 28-O-β-D-glucopyranoside (Mencherini et al., 2007) 3,23-Disulfate ester of 3β,23,29- trihydroxyolean-12-en-28-oic acid (Mencherini et al., 2007) 3,23-Disulfate ester of 2α,3β,23,29- tetrahydroxyolean-12-en-28-oic acid (Mencherini et al., 2007) 3,23-Disulfate ester of 2α,3β-23,29- tetrahydroxyolean-12-ene-28-oic acid, 28-Oβ-D-glucopyranoside (Tantry et al., 2014) 23-Monosulfate ester of 2α,23-dihydroxyurs12-ene-28-oic acid, 3-O-β-D- glucopyranoside (Tantry et al., 2014)
Polyhenolics -Phenolic acids		Rosmarinic acid (Ibragic et al., 2014) Caffeic acid (Ibragic et al., 2014) Protocatechuic acid (Ibragic et al., 2014) Cinnamic acid (Ibragic et al., 2014) Chlorogenic acid (Ibragic et al., 2014) Gallic acid (Ibragic et al., 2014) Ferulic acid (Arceusz et al., 2015) Ellagic acid (Arceusz et al., 2015) *p*-Coumarinic acid (Arceusz et al., 2015) Salvianolic acid (Barros et al., 2013)
Flavonoids -Flavones		Luteolin (Mencherini et al., 2007) Apigenin (Mencherini et al., 2007)
-Flavanones		Hesperidin (Pereira et al., 2014; Shakeri et al., 2016) Hesperetin (Pereira et al., 2014; Shakeri et al., 2016) Naringin (Pereira et al., 2014; Shakeri et al., 2016) Naringenin (Pereira et al., 2014; Shakeri et al., 2016)
-Flavanols		Catechin (Patora and Klimek, 2002; Shakeri et al., 2016) Epicatechin (Patora and Klimek, 2002; Shakeri et al., 2016) Routin (Patora and Klimek, 2002; Shakeri et al., 2016)
-Flavonols		Quercetin (Arceusz et al., 2015) Myricetin (Arceusz et al., 2015) Quercitrin (Patora and Klimek, 2002; Mencherini et al., 2007; Shakeri et al., 2016) Rhamnocitrin (Patora and Klimek, 2002; Mencherini et al., 2007; Shakeri et al., 2016) Isoquercitrin (Patora and Klimek, 2002; Mencherini et al., 2007; Shakeri et al., 2016)

## Traditional Use of MO

Lemon balm has been extensively used in traditional medicine for different medical purposes due to its history dating back more than 2,000 years ago (Zarei et al., 2015). The father of pharmacology Dioscorides was the first who mentioned this herb in his Pharmacopeia of medicinal plants, De Materia Medica. Since then, the healing properties of MO have been noted in many other medical books. In addition to its large use in traditional medicine, MO is also used in the food industry as well as in aromatherapy due to its fresh smell. It is noteworthy that only aerial plant parts are traditionally used, whereas its roots attract less attention (Shakeri et al., 2016). Several studies pointed out different effects of lemon balm such as sedative and mild hypnotic (Nour Eddine et al., 2005; Chen et al., 2006; Rasmussen, 2011), hypoglycemic, hepatoprotective, antibacterial, anti-inflammatory, antioxidant, antiviral, antispasmodic, and neuroprotective (Zarei et al., 2015; Moacǎ et al., 2018). Nevertheless, there is evidence that indicates the cytotoxic effects of MO extract (MOE) on breast (Zarei et al., 2015) and colon carcinoma (Weidner et al., 2015). Because of the ability to induce apoptosis and inhibit colon cancer cell proliferation, it has been proven that RA from MOE is responsible for its antimigratory effect on colon cancer cells (Encalada et al., 2011).

Since it has been well known that hypertriglyceridemia is one of the most important factors for CVD development, a strong effort has gone into prevention strategies including a phytochemical diet. Additionally, studies showed that daily drinking of MO tea may ameliorate metabolic parameters through reduction of serum lipid concentration and lipid peroxidation. Although the lipid-lowering mechanism of lemon balm remains unclear, it is proposed that the presence of quercetin is responsible for this pharmacological effect (Jun et al., 2012). In addition, Bolkent et al. (2005) reported that besides the reduction of total cholesterol (TC), MOE also possesses the ability to increase glutathione (GSH) levels in the liver tissue. However, the hypolipidemic effects of MOE are likely related to its powerful antioxidant properties due to lemon balm containing phenolic compounds, as the most important antioxidant agents (Zarei et al., 2015). It is assumed that the presence of RA, a derivate of phenylpropanoid, is associated with its antioxidant properties that are up to 10 times stronger than the antioxidant action of vitamin C (Shakeri et al., 2016). Furthermore, lemon balm extract acts as a potential scavenger of both synthetic and natural free radicals during their early or later stages of formation (Pereira et al., 2009). Compounds of MO possess the ability to bind to acetylcholine, as well as inhibit enzyme acetylcholinesterase, which in turn leads to improved cognitive function such as memory (Rostami et al., 2010). Finally, the researchers stand out that MOE prevents diseases that are related to oxidative stress including diabetes, CVDs, or neurodegenerative disorders such as Parkinson and Alzheimer diseases (Pereira et al., 2009). Daily intake of MO at low doses improves glucose tolerance and adjusted gene expression that is involved in hepatic gluconeogenesis. All together, these mechanisms contribute to the hypoglycemic properties of this herb (Chung et al., 2010). When it comes to anxiolytic effects of MOE, the results of several studies implicate that lower doses of aqueous extract have anxiolytic properties; whereas applied in higher doses, it has a sedative effect (Zarei et al., 2015). In some European countries, this herb is traditionally used for relaxation, especially when a disturbance occurs in the first stage of sleep. Also, it is found that MOE can reduce agitation, as well as muscle tone in patients with Alzheimer disease (Soulimani et al., 1991). Traditionally, use of lemon balm in mentally ill patients results in significant improvement in irritability, insomnia, headaches, and heart disease (Ulbricht et al., 2005). All of these beneficial effects can be attributed to the presence of significant amounts of rosmarinic, oleanolic, ursolic acid, and triterpenoids in the herb, assuming that all of these active principles can inhibit γ-aminobutyric acid (GABA) transport activity and increase the level of this neurotransmitter in the brain (Awad et al., 2007; Ibarra et al., 2010).

The use of herbs to relieve pain has a long history in the world of medicine. The fact that the essential oil of lemon balm has anti-inflammatory properties supports the traditional use of this herb in the treatment of numerous diseases associated with inflammation or pain (Bounihi et al., 2013). Literature data indicate that anti-inflammatory and antinociceptive effects of MO are contributed to RA, as well as to flavonoids and terpenoids present in the extract. Although acute analgesic property of MOE does not differ from morphine or aspirin, its chronic analgesic effect is less effective. On the other hand, active principles in the MO inhibit the monoamine oxidase enzyme and thus prevent degradation of catecholamines, but also inhibit cyclooxygenase enzyme and decrease prostaglandins and inflammatory cytokine production as in response to inflammation stimuli. In this way, the traditional use of MO is absolutely justified in the treatment of inflammatory diseases (Zarei et al., 2015). There is also evidence regarding the antiviral effects of MOE at a very early stage of the infection with respect to herpes simplex virus type 1 (HSV-1) (Miraj et al., 2017). Based on the results of an *in vitro* study, it is suggested that MO possesses a high virucidal effect against HSV-1 even at a very low concentration. It is assumed that RA contributed to this antiviral effect (Astani et al., 2012). Lemon balm oil affects virus before adsorption, but it is not effective after virus penetration into the host cell. Finally, taking into account that lemon balm essential oil is able to penetrate the skin, thanks to its lipophilic nature, it is worth noting that MO oil should be suitable for topical treatment of herpetic infection (Wolbling and Leonhardt, 1994).

## Cardiac Effects (Arrhythmia, Ischemia–Reperfusion Injury, Myocardial Infarction)

### Preclinical Studies

Because of the long known antiarrhythmic effect of lemon balm and its traditional use for heart palpitations, a wide spectrum of *in vitro*, *in vivo*, and *ex vivo* animal studies were conducted in order to elucidate the mechanisms of this therapeutic property (Table 2).

**TABLE 2:** Cardiovascular effects of *M. officinalis.*

Study	Effect	Model	Material	*M. officinalis* formulation	Dosage	Mechanism
Gazola et al. (2004)	↓Cardiac rate	Langendoff technique *ex vivo* isolated rat heart	*Rattus norwegicus albinus*	Aqueous extract	0.038, 0.38, 3.8, and 38 mg acutely heart perfusion (0.5 mL)	Activation of cardiac M2 receptors Blockage of Ca^2+^ channels
Joukar et al. (2014)	↓Ventricular arrhythmias Blood pressure	LAD model of *in vivo* myocardial I/R injury	Wistar albino rats	Aqueous extract	50, 100, 200, and 400 mg/kg intraperitoneally	Activation of cardiac M2 receptors Antioxidant effect of phenolics present in MOE
Joukar and Asadipour (2015)	Prolonged QTc JT and TpTe interval	ECG	Wistar albino rats	Aqueous extract	50, 100, and 200 mg/kg	Blockage of K^+^ channels and slowing ventricular conductivity
Joukar et al. (2016)	↓Blood pressure ↓Heart rate by lower doses of MOE	Isoproterenol-induced myocardial infarction	Wistar albino rats	Aqueous extract	50, 100, and 200 mg/kg *per os* 7 days	↓Oxidative stress ↓MDA
Sedighi et al. (2019)	↓Infarct size ↓Ventricular arrhythmias ↓Oxidative stress	LAD *In vivo* regional I/R injury	Sprague–Dawley rats	Ethanolic extract	25, 50, and 100 mg/kg *per os* 14 days	↓MDA, ↑SOD Free radical–scavenging of phenolic compounds
Hamza et al. (2016)	Dose-dependent amelioration of doxorubicin-induced cardiotoxicity	Doxorubicin-induced cardiotoxicity	Wistar albino rats	Ethanolic extract Macerate	250, 500, and 750 mg/kg *per os* 10 days	↓Oxidative stress (↓MDA, ↓TOC, ↓protein carbonyl, ↑SOD) antioxidant capacity of MOE anti-inflammatory (↓NF-κB, TNF-α, and COX-2 gene expression) antiapoptotic effect (↓Bax, ↓caspase-3)
Akhondali et al. (2015)	↓Heart rate ↓Incidence of ventricular arrhythmias	CaCl_2_-induced arrhythmia	Sprague–Dawley rats	Ethanolic extract Macerate	100 and 200 mg/kg Gastric gavage 14 days	Antioxidant effect of phenolics present in MOE
Safaeian et al. (2016)	Cytoprotection Only via doses 100–500 μg/mL	H_2_O_2_-induced toxicity in vascular cells	*In vitro* HUVECs	Hydroalcoholic extract Macerate	25, 50, 100, 250, and 500 μg/mL	Antioxidant effects of rosmarinic acid and other phenolics present in MOE
Ersoy et al. (2008)	Dose-dependent vasorelaxant effect Of the extract and isolated rosmarinic acid	Rat isolated thoracic aorta	Wistar albino rats	Aqueous extract Infuse	0.001–1 mg/mL dilution in Krebs–Henseleit solution	NO pathways and prostacyclin pathways involvement
Alijaniha et al. (2015)	↓Incidence of heart palpitation episodes	Benign heart palpitation	Healthy human volunteers (*n* = 71)	Lyophilized aqueous extract	500 mg *per os* 2 × a day, for 14 days	—

One of the initial researches regarding this subject was conducted in the isolated rat hearts by using Langendorff technique. It has been revealed that acute application of aqueous MOE in the isolated rat heart had negative chronotropic effect, without the alteration of contractile force. Multiple mechanisms triggered by plant’s alkaloids may be considered responsible for these effects, such as stimulation of muscarinic receptors in the heart and/or by voltage-dependent calcium channel voltage blockage inducing bradycardia (Gazola et al., 2004). However, Joukar et al. did not confirm this effect, possibly due to different model of myocardial ischemia–reperfusion injury (I/R) injury, *in vivo* [ligation of left descending coronary artery (LAD)] vs. previously used *ex vivo* isolated rat heart model. They reported a mild antiarrhythmic effect of aqueous MOE compared to standard antiarrhythmic drug amiodarone, with multimodal mechanism of action. MOE application–induced slower heart electrical conduction through partial PR and QTc prolongation in electrocardiogram (ECG), decreased susceptibility to reperfusion–associated ventricular arrhythmias (fibrillation), and an improvement in blood pressure especially when given in a dose of 200 mg/kg intraperitoneally. All of these changes were of a less extent relative to amiodarone and attributed to both antioxidant effects of phenolic compounds in lemon balm and stimulation of muscarinic receptors, acetylcholine K^+^ channels, which may be connected with slower conduction in the heart and prolonged PR interval (Joukar et al., 2014). Lemon balm also contains flavonoids, phenolic acids, terpenes, RA, and caffeic acid, all of which can have antioxidant effects. One year later, the same research group evaluated the effect of 8-day consumption of water MOE in three different doses (50, 100, and 200 mg/kg) on ECG and electrophysiology of the rat heart. They reported that MOE consumption is associated with prolonged QRS, QTc, JT, and TpTe interval in a dose-dependent way with no alterations of RR interval, PR interval, amplitudes of ECG waves (P duration, P amplitude, Q amplitude, R amplitude, S amplitude, T amplitude, and ST height), heart rate, and blood pressure. QRS prolongation implies the possible antiarrhythmic effect of MOE by slowing ventricular conductivity, similarly to class I antiarrhythmics. On the other hand, QTc and JT prolongation induced by MOE is also characteristic for class III antiarrhythmics via blockage K^+^ channels (Joukar and Asadipour, 2015). Nonetheless, both of these alterations could potentially turn into proarrhythmogenic if MOE is applied in higher doses, subsequently inducing early after-depolarizations that might increase the risk of ventricular extrasystoles, reentry phenomenon, and torsade de pointes arrhythmia (Gupta et al., 2007). Cardiac effects of MOE were also evaluated in a well-established model of isoproterenol-induced myocardial infarction, emphasizing the importance of MOE dosage. They emphasized that lower doses of MOE (50 and 100 mg/kg) provide better cardioprotection by reducing the arterial pressure and heart rate and also by improving redox state via a decline in malondialdehyde (MDA) levels. More importantly, this study added new facts regarding the safety of MOE, as high dose of MOE (200 mg/kg) led to intensification isoproterenol-induced cardiac injury under myocardial ischemia conditions, as a consequence from increasing of cardiac contractility, increasing of myocardial oxygen demand, and hence more risk of cardiac injury (Joukar et al., 2016).

Novel study pointed out potent cardioprotective effect of ethanolic MOE against cardiac I/R and subsequent arrhythmias in *in vivo* rat model of regional heart ischemia (LAD model) (Sedighi et al., 2019). Namely, 2-week oral application of this extract led to infarct size reduction, decrease of ventricular tachycardia, and ventricular ectopic beats episodes, stabilizing the ST segment changes and QTc shortening, and increased the R and T wave amplitudes and the heart rate during ischemia. Antiarrhythmic effects were dose-dependent, 100 mg/kg being the most effective dose of MOE. Cinnamic acid, as a major phenolic compound of this extract, was considered to be responsible for these effects via amelioration of oxidative stress. Rich phenolic content of lemon balm may induce cardioprotection in several mechanisms as reducing agents, free radical scavenging, and potential chelation of pro-oxidant metals (Miraj et al., 2017; Sedighi et al., 2019). Consistent results regarding antiarrhythmic effects of ethanolic MOE were also confirmed in CaCl_2_-induced arrhythmia model in Sprague–Dawley rats. Two-week consumption of MOE decreased the heart rate and the incidence of ventricular tachycardia, ventricular fibrillation, and ventricular premature beats, especially in a higher dose of 200 mg/kg (Akhondali et al., 2015). These ECG changes were linked with antioxidant effects of polyphenolics and vasorelaxant effect of monoterpene citral from MOE (Wolbling and Leonhardt, 1994; Gazola et al., 2004; Gupta et al., 2007; Astani et al., 2012; Devi et al., 2012; Joukar et al., 2014, 2016; Akhondali et al., 2015; Joukar and Asadipour, 2015; Miraj et al., 2017; Sedighi et al., 2019). Taking the fact that the methanolic extracts of MO were previously reported to induce vasorelaxation through blockage of the Ca^2+^ channels and that these channels play an important role in pacemaker currents in atrioventricular node, this may be the mechanism of MOE’s heart rate–slowing property (Devi et al., 2012). Capability of ethanolic MOE to alleviate the most frequent cytotoxic drug–induced cardiotoxicity and doxorubicin was thoroughly investigated by Hamza et al. (2016) Namely, they proved dose-dependent ability of MOE to preserve cardiac function and morphology in well-established doxorubicin-induced cardiotoxicity rat model via oxidative stress modulation and amelioration of both inflammation and apoptosis in rat heart. Surprisingly, optimal cardioprotection was induced by MOE in a dose of 750 mg/kg (Hamza et al., 2016), suggesting great therapeutic index of MOE regarding safety and efficacy, as most previous studies that investigated the cardiovascular effects of MOE used lower doses such as 50, 100, and 200 mg/kg (Joukar et al., 2014, 2016; Sedighi et al., 2019). It is widely known that anthracycline antibiotic doxorubicin, commonly used for various malignancies, causes deleterious effects on the myocardium, leading to cardiomyopathy and/or subsequent heart failure, thus limiting its successful usage in oncology patients. In recent years, various studies unraveled the mechanisms of this cardiotoxicity including reactive oxygen species (ROS)–induced oxidative damage, interfering with inflammation and apoptosis of cardiomyocytes (Zhao and Zhang, 2017; Renu et al., 2018; Songbo et al., 2019). Ethanolic MOE in the dose of 750 mg/kg abolished all of these harmful effects of doxorubicin via amelioration of oxidative stress through increase in antioxidant capacity [superoxide dismutase (SOD)] and lipid peroxidation decrease (MDA reduction). Additionally, MOE abrogated inflammation by downregulating the expression of nuclear factor κ light-chain enhancer of activated B cells (NF-κB), tumor necrosis factor α (TNF-α), and cyclooxygenase 2 (COX-2) genes and showed antiapoptotic activity through decrease in Bax and caspase-3 expression (Hamza et al., 2016). What is more, MOE prevented the leakage of cardiac enzymes [creatine kinase myocardial band (CK-MB), troponin I, and troponin T] and preserved the morphology of the myocardium histopathologically when administered as adjuvant therapy with doxorubicin. Again, all of these beneficial effects of MOE extract are most likely induced by synergistic interactions of phenolic compounds and other triterpene acids of MOE.

### Clinical Studies

Although clinical data regarding cardiovascular benefits of MO and its different formulations are limited, there are some encouraging results from novel clinical trials (Table 2). Namely, double-blind, randomized, placebo-controlled trial of efficacy and safety was conducted in order to investigate and justify traditional use of MO for heart palpitations. It was found that 2 weeks of consumption lyophilized aqueous MOE (500 mg; two times per day) reduced heart palpitation episodes by 36% compared to 4.2% reduction in the placebo group. Additionally, no side effects were noted in the subjects who finished the study (*n* = 55) (Alijaniha et al., 2015). Other available clinical trial results were focused on the influence of MO on cardiometabolic parameters, such as lipid status, glycemia, and inflammatory status as factors promoting cardiovascular risk in diabetic patients (Asadi et al., 2018, 2019). Both trials came to similar results that MO application improves lipid ratios (Alijaniha et al., 2015), high-density lipoprotein (HDL), triglycerides (TGs), high-sensitivity C-reactive protein (hs-CRP) levels, systolic blood pressure (Asadi et al., 2019; Nayebi et al., 2019), and diastolic blood pressure (Ibragic et al., 2014), thus preventing cardiovascular risk in this patient population. It is noteworthy that these beneficial effects were achieved by a higher dose of MO (700 mg, 2 × a day, *per os*) and longer (3 months) usage (Asadi et al., 2018, 2019) than for heart palpitations (Zhao and Zhang, 2017). Nonetheless, no dose-dependent adverse reactions were reported (Asadi et al., 2018, 2019). Beneficial effects of hot water MOE were highlighted by Yui et al. (2017), in the aspect of the reductions in brachial-ankle pulse wave velocity, which reflects arterial stiffness in healthy adult subjects.

## Vascular Effects of MO

Vasorelaxant effects of aqueous MOE on isolated thoracic aortic rings were first revealed and reported by Ersoy et al. (2008) They proved that aqueous MOE induces endothelium-dependent vasorelaxation, and that possible mechanism of this effect involves mainly nitric oxide (NO) pathway, but also EDHF (endothelium-derived hyperpolarizing factor) and prostacyclin pathways. It may be assumed that this vasorelaxation may be induced by monoterpene component citral and mediated by aortic Ca^2+^ channel blockage also, besides NO pathway involvement. The reason for this is that it has been shown that methanolic MOE exhibits vasorelaxation by the Ca^2+^ channel blockage (Devi et al., 2012). Additionally, as RA was shown to be the most abundant component of aqueous MOE, it has been tested on isolated rat thoracic aorta. In that way, dose-dependent vasorelaxant effect of RA was proven (Ersoy et al., 2008).

Antioxidant and cytoprotective effects of hydroalcoholic MOE were also confirmed at the cellular level in a model of H_2_O_2_-induced oxidative stress in HUVECs (human umbilical vein endothelial cells) vascular cells, almost a decade later. It was found that hydroalcoholic MOE protects the vascular cells against H_2_O_2_-induced toxicity only when applied at higher concentrations (100–500 μg/mL) as evidenced by FRAP assay, FOX1 method, and MTT assay. Importantly, MOE was investigated for an eventual toxicity on HUVECs, and it was proven that MOE did not change cell viability 24 h after the exposure, except in the case of the highest concentration of MOE (1,000 μg/mL) (Yui et al., 2017). These cytoprotective effects of MOE are linked with the main component of MOE-RA, which provides vascular cytoprotective effects via several mechanisms: antioxidant (superoxide scavenging, inhibition of low-density lipoprotein (LDL) oxidation in human aortic endothelial cells) and antiangiogenetic (reduction in the expression of H_2_O_2_-dependent endothelial growth factor and release of interleukin 8 (IL-8) from endothelial cells and inhibition of the proliferation, migration, adhesion, and tube formation of HUVECs) (Pearson et al., 1997; Huang and Zheng, 2006; Safaeian et al., 2016).

## Effect of MO On Inflammatory Heart Diseases

Therapeutic effects of MO in inflammatory heart disease have not been investigated to date. Nonetheless, over a decade ago, pioneer evidence regarding the influence of water MOE on immune response in mice was reported (Drozd and Anuszewska, 2003). Additionally, several phenolic components present in MOE were shown to be beneficial in animal models of myocarditis, such as flavonoids quercetin, luteolin, and apigenin as they modulate immune response, decrease inflammation, and subsequently may suppress cardiac tissues remodeling, which occurs in dilative cardiomyopathy characteristic for chronic phase of myocarditis (Milenković et al., 2010; Zhang et al., 2016; Wu et al., 2020). Namely, chronic 3-week quercetin supplementation showed dose-dependent protective effect in experimental autoimmune myocarditis rat model through suppression of proinflammatory cytokines TNF-α and IL-17 and up-regulation of IL-10 (Milenković et al., 2010). Apigenin treatment ameliorated experimental autoimmune myocarditis in BALB/c mice by modulating T_H_1/T_H_2 balance through downregulation of T_H_1 cytokines (TNF-α, IL-2, and interferon γ) and up-regulation of T_H_2 cytokines (IL-4, IL-10) (Zhang et al., 2016). On the other hand, luteolin was proven to inhibit coxsackievirus B3 (CVB3) replication via depressing the phosphorylation of p38 MAPK and JNK MAPK and inhibiting NF-κB nuclear translocation and further attenuation of the inflammatory cytokine expression in CVB3-infected cells. Thus, luteolin may be beneficial in CVB3-induced myocarditis by inhibiting heart inflammation (Wu et al., 2020). Having this in mind, MO represents a great phenolic mixture with high potential as an adjuvant therapy for inflammatory heart disease, which should be clarified in future studies.

## Cardiometabolic Effects of MO

Cardiometabolic diseases have become the leading cause of death in the modern era worldwide. The meaning of this term has been expanded, and nowadays, it refers to CVD, diabetes mellitus, and chronic renal failure (de Waard et al., 2019). Etiology is mostly explained by unhealthy sedentary lifestyle, lack of physical activity, inadequate diet, and smoking. A wide spectrum of risk factors that may lead to progression of cardiometabolic disease, including insulin resistance; increased TC, TGs, and LDL; and decreased HDL. In that sense, the plant extract supplementation may be of great importance as a prevention strategy for cardiometabolic diseases, as polyphenol-rich plants are recognized as safe and supplements with great benefit on the health, especially on lipid status and glycemia (Cicero et al., 2017). Bolkent et al. (2005) were among the first to provide evidence about hypolipidemic properties of MOE. Namely, aqueous MOE when applied in a dose of 2 g/kg induced significant decrease in serum cholesterol and total lipids in high-fat diet model of hyperlipidemia in rats (Bolkent et al., 2005; Heshmati et al., 2020). Essential oil of MO (12.5 μg/d *per os*) was also shown to possess hypolipidemic properties via reduction of plasma TG in apolipoprotein E (ApoE) transgenic mice (Jun et al., 2012). Possible explanation for these hypolipidemic effects may lay in synergistic action of MOE active compounds, especially rosmarinic, ursolic, and oleanolic acids on serum lipids (Yuliang et al., 2015; Chen et al., 2017; Guo et al., 2020). Exact mechanisms are still not completely clear, but it is assumed that decreased expression of sterol regulatory element-binding protein-1c and its associated genes lead to decreased hepatic synthesis of fatty acids (Jun et al., 2012).

The usage of MO in cardiometabolic disease has been thoroughly explored in meta-analyses by Heshmati et al. (2020), and it seems that MO application most likely lowers TC and SBP, according to several studies results. Because of its strong antioxidant activity, MOE may be useful in treating or preventing dyslipidemia and possibly subsequent atherosclerotic process. This hypothesis was confirmed in several experimental studies and clinical trials (Bolkent et al., 2005; Jun et al., 2012; Weidner et al., 2014; Jandaghi et al., 2016; Asadi et al., 2018; Javid et al., 2018; Heshmati et al., 2020). In order to achieve these effects in human, attention should be given to optimization of MOE dosage. LDL-lowering effects of MO were reported when applied in a dose of 1,000 mg three times daily *per os* for 2 months in hyperlipidemic patients (Jun et al., 2012). Similarly, improvement of lipid status via Apo A-I, Apo B/Apo A-I, and lipid ratios was proven in type 2 diabetic patients treated with hydroalcoholic MOE 700 mg two times daily for 12 weeks (Asadi et al., 2018), whereas in patients with stable angina pectoris, higher doses (3 g/d) are needed to provide improvement in lipid profile (TC, LDL, HDL, TG), MDA, hs-CRP, and paraoxonase 1. Taking these aforementioned facts, the usage of MO may be a safe and useful way to improve metabolic markers and reduce the risk of cardiovascular consequences.

## Safety of MO and Possible Drug Interactions

The traditional usage of MO is generally considered to be well tolerated and safe, as it is assigned to the Food and Drug Administration GRAS (Generally Recognized as Safe) list in the United States. Up until now, no serious adverse effects have been reported following lemon balm usage. Because of insufficient information about lemon balm usage in sensitive populations such as pediatrics or during pregnancy or lactation, theoretically, in these cases it may be marked as possibly unsafe. *Per os* usage of MO has been reported to be well tolerated, with no adverse events when taken at less than 8 weeks. To date, sporadic minor side effects have been reported such as electroencephalographic changes in doses of 1,200 mg, reduced alertness with caution to driving in dose of 900 mg, possible increase of intraocular pressure, thyroid hormone inhibition, palpitations, headache, diarrhea, and vomiting (Ulbricht et al., 2005). Increased appetite was also reported as a side effect in a clinical trial investigating MO effects on heart palpitations (Alijaniha et al., 2015). On the other hand, topical administration of MO preparations might cause local redness, burning sensation, or irritation. It is considered that MO has no toxic effects on the liver, as proven in cell cultures (Carocho et al., 2015). Toxic effects of MO essential oil on the stomach, duodenum, liver, and kidneys have been described when applied in a dose of 1 mg/kg *per os*. The calculated LD50 (median lethal dose) for BALB/c mice is 2.57 g/kg, and this is thought to be moderately toxic for human usage (Stojanović et al., 2019). These findings are contrary to the previous study by Bounihi et al. (2014), which reported absence of toxicity after 2 g/kg MO essential oil of MO.

It is familiar that patients tend to use herbal products in the purpose of healing and curing considering it completely safe regardless of pharmacotherapy they are assigned to. As MO is most commonly used as a mild sedative because of its calming effects, to date, the only described interactions of MO refer to synergism with sedatives and barbiturates and inhibition of SSRIs (selective serotonin reuptake inhibitors) (Posadzki et al., 2013). MO exerts its therapeutic properties via inhibition of acetylcholinesterase activity and GABA transaminase, so it potentiates the effects of drugs acting in the same way. Thus, the usage of MO OTC preparations should be careful and monitored or advised by a pharmacist in patients using these drugs. This is because there is a possibility that MO potentiates the depressing effect on the central nervous system. Theoretically, MO may also interact with thyroid hormone therapy in the conditions of hypothyroidism, because of its ability to inhibit the thyroid hormone receptors (Ulbricht et al., 2005). Identification of MO interactions with cardiovascular drugs is of great significance as few years ago, a descriptive study employing cardiopathy patients on anticoagulation therapy, well known to be prone to dangerous drug and/or herb interactions, reported that high percentage of these patients self-used herbal medications, with MO being on the third place by frequency (Leite et al., 2016). There is limited amount of data regarding the possible interactions of MO or its components with cardiovascular drugs. Interestingly, it was recently reported that the most abundant component of MO, RA, may weakly inhibit the two isoforms of cytochrome P450 oxidase, CYP2C19, and CYP2E1 and moderately competitively inhibit UGT1A1, UGT1A6, and UGT2B7 (Kim et al., 2019). This fact may be important in predicting possible pharmacokinetic interactions with cardiovascular drugs metabolized by the mentioned isoforms such as carvedilol, simvastatin (UGT1A1 substrates) (Marques and Ikediobi, 2010), dabigatran etexilate, simvastatin, and fluvastatin (UGT2B7 substrates) (Drug Bank, 2021). Of course, these claims remain theoretical, as no preclinical or clinical studies evaluated these interactions. Another component of MO, flavonoid quercetin, was proven to interact with digoxin in terms of raising its bioavailability, which may result in the potentially harmful effect of this drug, well known to possess narrow therapeutic index (Wang et al., 2004).

Data regarding the aspects of MO and its active compounds in different formulations such as adverse effects of the extracts and potential interactions or synergistic effects, especially in polyherbal preparations, with standard cardiovascular medications, are still limited, thus calling for future studies especially in humans.

## Therapeutic Potential of RA

RA [*Rosmarinus officinalis* (RO)], as an isolated compound, was mentioned for the first time by Scarpati and Oriente in 1958 and carries the name in accordance with the plant from which it was first isolated, RO. It is a water-soluble ester of caffeic acid and 3,4-dihydroxyphenyl lactic acid and is prevalent in a wide range of plants. Additionally, it is a predominant phenol of several medicinal plants that belong to the Lamiaceae family, such as rosemary, sage, basil, mint, and lemon scent plants-emon balm (MO) (Prasannarong et al., 2019; Ghasemzadeh Rahbardar and Hosseinzadeh, 2020). It has been reported that the presence of RO in medicinal plants has generated healthy and beneficial effects such as antioxidant, astringent, antimicrobial, anti-inflammatory, antiviral, antiangiogenic, antirheumatic, antiallergic, antidiabetic, antidepressant, and antitumor (Ferreira et al., 2013). However, reports on its activity in the cardiovascular system are scarce but showed that RO possesses cardioprotective effects.

RO was shown to reduce blood pressure in ways that could be related to inhibition and/or modulation of angiotensin-converting enzyme or promotion of NO production and downregulation of endothelin-1 production (Li et al., 2008; Karthik et al., 2011). The mechanisms involved in the endothelium-dependent vasodilating effect of RO remain unknown. Nevertheless, this effect can be explained by the fact that RO is a polyphenolic compound that has a vasodilating activity, through activation of NO pathway, EDHF, and prostacyclin (Fernandes et al., 2005; Ersoy et al., 2008). There is evidence claiming that RO can improve both cardiovascular and metabolic problems in hypertensive conditions, which is confirmed by the results of a study that evaluated the acute and chronic effects of RO in hypertensive rats. The acute and chronic treatment with RO reduced blood pressure and fasting plasma glucose levels, while the acute RA treatment increased skeletal muscle glucose transport activity along with extracellular signal–regulated kinase activity (Prasannarong et al., 2019). Cardioprotective effects of the treatment with RO were also shown in a model of doxorubicin-induced cardiotoxicity and pressure overload–induced cardiac dysfunction in mice. Possible explanation of these effects may be reflected in the fact that RA alleviates cardiomyocyte apoptosis via cardiac fibroblast (Zhang et al., 2018, 2019). Preconditioning with RO could strongly prevent cardiac hypertrophy, lipid peroxidation, weakness in myocardial contractility and relaxation, and stretch-induced arrhythmias after acute myocardial infarction induced by supramaximal administration of isoproterenol in rats. The cardioprotective effect of RO, which was reflected through the reduction of cardiac hypertrophy, may be related to the inhibition of the isoproterenol-induced cardiac hyperactivity, resulting in inhibition of oxidative stress and lipid peroxidation (Javidanpour et al., 2018). In addition, 2 week application of RO exerted the beneficial effects on heart electrophysiology of rats with myocardial infarction. Namely, RO induced QRS complex voltage increment and dramatic lowering of ST elevation, especially via the highest dose applied (30 mg/kg) compared to non-treated animals with myocardial infarction. These results suggest the protective effect of RO on plasma membrane. RO pretreatment diminishes almost all harmful heart alterations in isoproterenol-induced myocardial infarction. This refers to antioxidants boosting antioxidant activity and up-regulation of gene expression of RyR2 and SERCA2, which are involved in Ca^2+^ homeostasis and possibly through RA antiadrenergic effect (Javidanpour et al., 2017). Considering these facts, application of RO isolated from MO may also be a useful tool for cardioprotection. Nonetheless, the synergistic action of all compounds present in MOE may provide even better effects.

## Cardioprotective Mechanisms of Other MO Active Compounds

There are a vast number of studies claiming that triterpene compounds of MO ursolic and oleanolic acid are *per se* cardioprotective. For example, ursolic acid exerts cardioprotection in different ways. First, it was shown that ursolic acid possesses negative inotropic and dromotropic effect, thus inducing antiarrhythmic effect in rats (Seo et al., 2018). Additionally, it was shown that pretreatment with ursolic acid alleviates myocardial I/R, isoproterenol induces myocardial infarction (Radhiga et al., 2012), and doxorubicin induced cardiotoxicity (Mu et al., 2019) via several mechanisms: antiapoptotic effect on cardiomyocytes, anti-inflammatory effect, antioxidant effect through suppression of ROS formation, and enhancement of antioxidant protection [SOD, catalase (CAT), GSH] (Wang et al., 2018). There is also evidence that oleanolic acid could improve the release of NO from the endothelium and induce vasorelaxation, which is mediated by phosphoinositide-3-kinase–dependent phosphorylation of Akt-Ser473 followed by phosphorylation of eNOS-Ser1177 (Rodriguez-Rodriguez et al., 2008).

Besides thoroughly described phenolic acid most abundant in MO-RA, additional phenolic acids such as gallic acid, caffeic acid, ferulic acid, etc., may also contribute to cardioprotection induced by MO through several mechanisms. Gallic acid applied in a dose of 20 mg/kg was proven to reduce cardiac remodeling and hypertrophy via activation of autophagy, which subsequently causes the degradation of epidermal growth factor receptor, gp130, and CaNA and finally leads to the inactivation of their downstream pathways (ERK1/2, AKT, JAK2, STAT3, and NFATc1) (Yan et al., 2019). Gallic acid (13.1 mg/kg) was also reported to improve cardiac dysfunction and fibrosis in a model of pressure overload–induced heart failure via decreased expression of collagen type I and connective tissue growth factor (Jin et al., 2018). Ferulic acid application (20 and 40 mg/kg) was shown to protect the heart against doxorubicin-induced cardiotoxicity as proven by the decrease of natriuretic peptides atrial natriuretic peptide and brain natriuretic peptide, cardiac enzymes CK-MB and lactate dehydrogenase, decrease of proinflammatory cytokines IL-1β and IL-6, and increase of antioxidant GSH in the myocardium. It is suggested that ferulic acid acts as an antihypertensive because of the existing evidence about its ability to decrease systolic blood pressure, left ventricular diastolic stiffness, antioxidant protection enzymes SOD and CAT boosting, amelioration of kidney damage caused by hypertension, and vasorelaxant effect on isolated thoracic aorta (Alam et al., 2013).

 Flavonoid components such as quercetin, rutin, and hesperetin are widely investigated as cardioprotective agents, with its potency and efficacy varying on applied doses or plant they are isolated from. As they are present in different extracts of MO, they could also be responsible for cardiovascular effects of this plant, together with the others. To date, numerous beneficial effects of quercetin on cardiovascular system have been described including antihypertensive, antiatherosclerotic, anti-ischemic, anti-inflammatory, and antioxidant effects. Dozens of mechanisms are proven to be involved in these effects. First, increased bioavailability of NO and decreased NADPH oxidase and eNOS activity lead to vasodilation and angiotensin-converting enzyme inhibition, all of them enabling better blood pressure regulation. Second, decreased oxidative stress, through augmentation of antioxidant protection enzymes CAT, SOD, and GSH; decreased lipid peroxidation; and decreased oxidation of LDL providing antiatherosclerotic action. Third, antiapoptotic effect and decrease of proinflammatory markers make quercetin a great candidate for cardiovascular drug (Patel et al., 2018).

Nonetheless, there is no evidence about the cardiovascular effects of these pure compounds isolated concretely from MO. As the beneficial effects of the mentioned compounds highly depend on dose, frequency, administration route, and treatment duration, it remains a question whether application of themselves would exert the same effect when isolated from MO. It is the most probable that the MO-induced cardioprotection is mediated through various mechanisms of these compounds and its synergistic action.

## Conclusion

Although there is promising preclinical evidence regarding cardiovascular benefits of MOEs and essential oil application, there is an urgent need for clarification of the exact mechanisms of cardioprotection, safety, and pharmacokinetics of MO on cellular level, so the translation of these results into clinical practice could be possible. Additionally, optimization of dosage in human remains a challenge as reported cardiac benefits in animals are achieved with different doses (50–500 mg/kg), whereas improvement of cardiometabolic markers is achieved with much higher doses, which should be questioned in clinical practice. Given the fact that insufficiently is known regarding the possible interaction of MO formulations with frequently used cardiovascular drugs or other medicinal plants, future studies should be focused on these problems. Additionally, the usage of MO was investigated in various models of CVD; nonetheless, in inflammatory heart disease, it has not been investigated so far. Future studies should also focus on revealing the effects of MO in this pathology also, as there is evidence about its anti-inflammatory effects. This review provided detailed information regarding ethnomedical, preclinical, and possible clinical usage of MO in various cardiovascular entities.

## Author Contributions

IM and ND substantially contributed to the conception and the design of the manuscript. ND, MA, MR, and JJ wrote sections of the manuscript. IS, TNT, JJ, AS, and MT selected extracted relevant manuscript of this manuscript. VJ, SB, and IM reviewed and approved the final version. All authors had full access to data and critically revised and approved the manuscript for publication.

## Conflict of Interest

The authors declare that the research was conducted in the absence of any commercial or financial relationships that could be construed as a potential conflict of interest.

## Abbreviations

CAT: catalase
CK-MB: creatine kinase myocardial band
COX-2: cyclooxygenase 2
CVB3: coxsackievirus B3
CVDs: cardiovascular diseases
CYP: cytochrome P450 oxidase
ECG: electrocardiogram
EDHF: endothelium-derived hyperpolarizing factor
eNOS: endothelial nitric oxide synthase
FOX1: ferrous oxidation–xylenol orange
FRAP: ferric reducing ability of plasma
GABA: transaminase–γ-aminobutyric acid transaminase
GRAS: generally recognized as safe
GSH: glutathione
H_2_O_2_: hydrogen peroxide
HUVECs: human umbilical vein endothelial cells
IL-10: interleukin 10
IL-17: interleukin 17
IL-2: interleukin 2
IL-4: interleukin 4
IL-8: interleukin 8
JNK MAPK: c-JunN-terminal kinases mitogen-activated protein kinase
LAD: left descending coronary artery
LDL: low-density lipoprotein
MDA: malondialdehyde
MO: *Melissa officinalis*
MOE: *Melissa officinalis* extract
MTT: 3-(4,5-dimethylthiazol-2-yl)-2,5-diphenyltetrazolium bromide
NADPH: nicotinamide adenine dinucleotide phosphate
NF-κB: nuclear factor κ light-chain enhancer of activated B cells
NO: nitric oxide
OTC: over the counter
p38 MAPK: p38 mitogen-activated protein kinase
ROS: reactive oxygen species
SOD: superoxide dismutase
SSRIs: Selective serotonin re − uptake inhibitors
TNF-α: tumor necrosis factor α
UGT: uridine 5’-diphospho-glucuronosyltransferase.
